# Synchronized Expression of Two Caspase Family Genes, *ice-2* and *ice-5*, in Hydrogen Peroxide-Induced Cells of the Silkworm, *Bombyx mori*

**DOI:** 10.1673/031.010.4301

**Published:** 2010-05-08

**Authors:** Y. Sun, W. Wang, B. Li, Y. Wu, H. Wu, W. Shen

**Affiliations:** ^1^ Institute of Life Sciences, Jiangsu University, Zhenjiang 212013, China; ^2^ School of Life Sciences, Soochow University, Suzhou 215123, China

**Keywords:** apoptosis, ultraviolet irradiation

## Abstract

Caspase family proteins play important roles in different stages of the apoptotic pathway. To date, however, functions of *Bombyx mori* L. (Lepidoptera: Bombycidae) caspase family genes are poorly known. This paper focuses on the morphology, mitochondrial membrane potential, and expression profiles of two novel *B. mori* caspase family genes (*ice-2* and *ice-5*) in 3 µM hydrogen peroxide (H_2_O_2_) damaged *B. mori* cells, which were separated from the ovary of *B. mori*. In addition, comparisons were made between damage caused by H_2_O_2_ and by ultraviolet (UV) irradiation. The results showed that the potential change of the mitochondrial membrane occurred at 0.5 h after H_2_O_2_ stimulation, which was sooner than occurred in the UV treated model where the obvious decrease appeared at 6 h after stimulation. In addition, the total change in the potential of the mitochondrial membrane in H_2_O_2_ treated *B. mori* cells was larger than with UV treated cells during the whole process. Analysis of fluorescent quantitative real-time PCR demonstrated that *ice-2* and *ice-5* might be involved in both H_2_O_2_ and UV-induced apoptosis in *B. mori* cells. Notably, after exposure to H_2_O_2_, the expression patterns of *ice-5* were remarkably higher than those of *ice-2*, while the result was the opposite after exposure to UV irradiation. The data indicate that apoptosis induced by H_2_O_2_ was directly related to the mitochondrial pathway. The two isoforms of *B. mori ice* may play different roles in the mitochondrion associated apoptotic pathway in *B. mori* cells, and the apoptotic pathway in H_2_O_2_ induced *B. mori* cells is different from the UV induced apoptotic pathway.

## Introduction

As a member of the caspase (cys-teiny-laspartate specific proteinase) family, interleukin -1 beta-converting enzyme (ICE) was discovered in mammals and named caspase-1. It is considered the initiator in caspase-dependent apoptosis. ICE was identified as a CED-3-like protein in *Caenorhabditis elegans* (Yuan et al. 1993). In lepidopteran insects, *ice* was identified as a pro-death factor in the *Heliothis virescens* midguts developmental apoptotic process (Parthasarathy and Palli 2007). According to the reported sequences in GenBank, three silkworm *ice* homologs — *ice*, *ice-2* and *ice-5* — were described (Accession numbers: *ice*, AY885228; *ice-2*, DQ360829; and *ice-5*, DQ360830). In a previous study (Song et al. 2007) *ice-2* and *ice-5* were cloned with an open reading frame of 852 and 936 base pairs (bps), respectively.

Many agents that induce apoptosis are either oxidants or stimulators of cellular oxidative metabolism (Haddad 2004). H_2_O_2_ is a reactive oxygen species. In general, reactive oxygen species are harmful to living organisms because they tend to cause oxidative damage to proteins, nucleic acids, and lipids (Hermes-Lima and Zenteno-Savín 2002). They also can induce various biological processes (Suzuki et al. 1997) and have been proposed as common mediators for apoptosis (Haddad 2004). H_2_O_2_ is an oxidant that triggers caspase activation and subsequent apoptosis (Blackstone and Green 1999). Therefore, the oxidative damage model based on H_2_O_2_ could be efficient for elucidating the roles of *ice-2* and *ice-5* in H_2_O_2_ induced apoptosis. Kidd (1998) reported that H_2_O_2_-mediated caspase activation was dependent on the release of cytochrome *c* from mitochondria, suggesting a key role for this peroxide in mitochondrial permeability and leakage. Before the release of cytochrome *c* from the mitochondria, the mitochondrial membrane potential was lost (Twomey and McCarthy 2005).

This study attempted to characterize the genes of *ice-2* and *ice-5* in the early phase of H_2_O_2_ induced apoptosis and to observe morphological and mitochondrial membrane potential changes in cells of *Bombyx mori* L. (Lepidoptera: Bombycidae). Meanwhile, time course transcriptional profiles of the two genes were investigated by quantitative realtime PCR. This report will provide new insight into the function of ICEs in insects. Additionally, damage caused by H_2_O_2_ and UV irradiation were compared in this paper and may provide insight into the role of insect ICEs during the apoptosis processes.

## Material and Methods

### *B. mori* cell culture

*B. mori* ovary-derived cells that were a gift of Dr. Xiangfu Wu (Chinese Academy of Sciences, Shanghai Institute of Biochemistry and Cell Biology) were cultured in TC-100 insect cell culture medium (Gibco brand, Invitrogen, www.invitrogen.com) supplemented with 10% fetal bovine serum at 27° C. H_2_O_2_ was applied to the *B. mori* cells, which then were plated at a density of 2 × 106 cells in 6-well plates (Corning, www.corning.com). They were incubated for 3–5 days at 27° C, and then used for further studies.

### Hydrogen peroxide treatment

Apoptosis was induced in *B. mori* cells by exposure to different concentrations (0.09 – 90 µM) of H_2_O_2_, and the median lethal dose (LD_50_) was calculated. While incubating at the LD_50_ H_2_O_2_ concentration, *B. mori* cells were observed microscopically at specified intervals for the appearance of apoptotic bodies, and were collected at regular intervals.

### UV irradiation treatment

The cells, with a very thin layer of phosphate buffered saline were irradiated for 20 s under UVA and UVB lamps at different UV doses (50 - 5 mJ/cm^2^). The total dosage was measured by a radiometer (International Light, Inc., www.intl-lighttech.com) fitted with a UV detector. At the LD_50_ H_2_O_2_ concentration, LD_50_, *B. mori* cells were observed microscopically at specified intervals for the appearance of apoptotic bodies, and were collected at regular intervals.

### MTT assay for cell mortality

The 3-(4,5-dimethylthiazol-2-yl)-2,5-diphenyl tetrazolium bromide (MTT) assay was used to detect mortality and was carried out according to Fornelli et al. (2004). Five mg/ml MTT was dissolved in phosphate buffered saline, and 20 µl of this stock solution was added to the culture wells. The incubation time with MTT was 3 h at 27° C. The supernatant was removed, and 150 µl of dimethyl sulfoxide was added to each well before reading optical density at 580 nm with fluorescence spectromety (Spectra Max, Gemini EM, Molecular Devices, www.moleculardevices.com). Mortality = 1-viability.

### JC-1 assay for mitochondrial membrane potential

Change in the potential of the mitochondrial membrane was assessed in live *B. mori* cells by using the lipophilic cationic probe 5,5′,6,6′-tetrachloro-1,1′,3,3′-tetraethylbenzimidazolcarbocyanine iodine (JC-1) (Smiley et al. 1991). For quantitative fluorescence measurement, cells were rinsed once after JC-1 staining and scanned with fluorescence spectrometry at 485-nm excitation and 530 and 590 nm emission, to measure green and red JC-1 fluorescence, respectively. Each well was scanned at 25 areas rectangularly arranged in 5 × 5 pattern with 1-mm intervals and an approximate beam area of 1 mm^2^ (bottom scanning).

### RNA extraction

Total RNA was extracted from the collected cells using Trizol (Invitrogen) according to the manufacturer's protocol. Contaminated genomic DNA was removed by Rnase-free Dnase I (Promega, www.promega.com). The concentration of the RNA was assessed using the Genspec III spectrophotometer (Hitachi Genetic Systems, www.biospace.com), and the integrity of the RNA was assessed by running 2 µl of RNA on a 1% ethidium bromide/agarose gel. The RNA was stored at -70° C until needed.

### Reverse transcription

2 µ g DNase-treated RNA was reversetranscribed to single stranded cDNA in a 20 µl reaction containing 0.2 µmol/L oligo-dT, 0.5 mmol/L of each dNTP, 5 µl M-MLV 5 × reaction buffer, and 200 U M-MLV reverse transcriptase (Promega). The thermal cycling profiles were as follows: 65° C for 5 min, 37° C for 60 min, and 75° C for 5 min. The resultant cDNA was stored at -20° C until needed.

### Quantitative real-time PCR

Primers used for the real-time PCR amplification of *ice-2*, *ice-5* and *B. mori* actin were selected based on the sequences available in GenBank. Primers were designed for specific detection (for *ice-2* Forward: 5′ tctgttgacggttatctttc 3′ and Reverse: 5′ tattgttggtctcctgacat 3′; for *ice-5*, Forward: 5′ tgttgacgagcttgtgactc 3′ and Reverse: 5′ caccatcgtgatcatatgca 3′). Primers for *B. mori actin* A3 (Forward: 5′ atccagcagctccctcga gaagtc t 3′ and Reverse: 5′ acaatggagggacca gactcgtcgt 3′) were used as an endogenous reference gene in real-time PCR.

Real-time PCR amplifications were performed to examine the relative expression of *ice-2* and *ice-5* in treated *B. mori* cells in the sequence detection system (MX3000P, Stratagene, www.stratagene.com). Duplicates of 0.5 µl cDNA from each reverse transcription reaction were used as templates. The reactions were performed in a total volume of 50 µl using SYBR premix EXTaqTM perfect realtime kit (TaKaRa, www.takara-bio.com) as recommended by the manufacturer. The following MX3000P thermocycling program was used: denaturation program (3 min at 95° C), amplification and quantification program repeated 40 times (10 s at 95° C, 30 s at 58° C and 20 s at 72° C with a single fluorescence measurement), and melting curve program (55° C to 95° C with a heating rate of 0.1° C/s).

**Figure 1.**
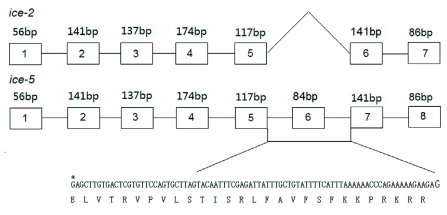
Exons of *ice-2* and *ice-5*. The number above the box shows the number of base pairs present in one exon. *ice-2* and *ice-5* are the same for the first 5 exons. The sixth and seventh exons of *ice-2* are the same as the seventh and eighth exons of *ice-5*. However, the sixth exon of *ice-5*, containing 84 bp, is absent in *ice-2*. DNA sequence and amino acid sequence are shown at the bottom of *ice-5*. The black star shows the base from the fifth exon of *ice-5.* High quality figures are available online.

Relative expression levels of *ice-2* and *ice-5* were calculated with the comparative Ct (2 -^ΔΔ^Ct) method. Means and standard errors for each time-point were obtained from the averages of three independent sample sets.

### Statistical analysis

Data are presented as the mean ± SD or mean ± SE of results of two or three separate experiments, as specified in the figure legends. Statistical significance was calculated (SPSS11.5, SPSS Inc., www.spss.com) with one-way ANOVA and one-sample T test. The p value lower than 0.05 was considered as significant.

## Results

### Sequence analysis of *ice-2* and *ice-5*

Sequence analysis suggested that *B. mori ice2* and *ice-5* resemble human caspase-3, which plays a role as an effector and depends on the release of cytochrome *c* from the mitochondrion. Interestingly, expression of the *ice* isoform was not detected in the previous study, since no copies of *ice* were Moreover, the isoforms, *ice-2* and *ice-5*, were transcribed from the same gene but spliced differently under UV irradiation, and they both have a QACRG active site that belongs to the caspase family (Song et al. 2007). Sequence analysis revealed that *ice-2* had seven exons, while *ice-5* had eight. The difference between the two genes was that *ice5* contained an extra exon with 84 bp, and the 28 amino acids are unique to *ice-5* (Figure 1).

**Table 1.**
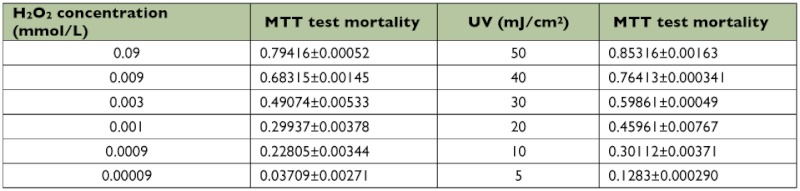
Dose-response obtained in response to H_2_O_2_ after 12 h of incubation and evaluated by MTT-Colorimetric assay

**Figure 2.**
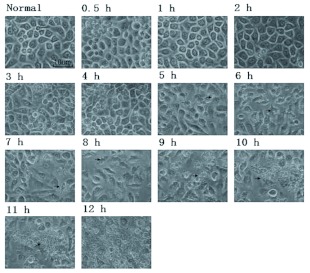
Progression of Bombyx mori cell apoptosis after H_2_O_2_ stimulation. Morphological changes in *B. mori* cells were observed from 0.5 to 12 h. Normal *B. mori* cells were used as a control. The numbers at the top of the panels show the time stage of the *B. mori* cell culture after H_2_O_2_ stimulation. The black arrow indicates typical morphology of the cell in the relevant stage. From 0.5 h to 4 h, few changes in morphology took place. At 5 h, spike-like membranes protruded from several cell membranes. From 6 h to 8 h, the cells became slender, and the spike-like membranes were still there. At 9 h, vesicles appeared, and the cells started to change shape. From 10 h to 12 h, the vesicles increased, and the cells became round. The photos were taken at 200× magnification.

### LD_50_ values for H_2_O_2_ and UV irradiation that induce cell apoptosis

Apoptosis was induced in *B. mori* cells by exposure to different concentrations (0.09 – 90 µM) of H_2_O_2_, and the LD_50_ value was calculated using the MTT assay. The same test was repeated with UV irradiation. Table 1 shows that the best concentration of H_2_O_2_ was 3 µM because mortality (49.074%) of 3 µM-treated *B. mori* cells was nearest to LD_50_. The best dose of UV irradiation was 20 mJ/cm^2^, with a mortality rate of 45.961%, which was the nearest to LD_50_.

### Morphological change of cells after H_2_O_2_ stimulation

Using a microscope, *B. mori* cells were observed after H_2_O_2_ stimulation at regular intervals from 0 to 12 h. As time passed, the morphology of the cells changed. However, in the first 4 h after stimulation, there were a few cells that had different morphology from the normal cells (Figure 2). Then some cell membranes wrinkled and the cells became smaller than normal cells by 5 h after stimulation. By 6 h after stimulation, wrinkling was more obvious. Bubble-like bodies appeared around wrinkled cells at 9 h post-stimulation. Vesicles formed in cell membranes, and apoptotic bodies were observed from the 10 h to 12 h phase.

**Figure 3.**
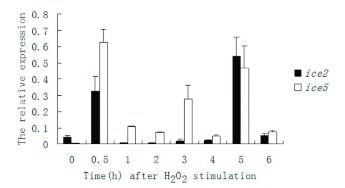
Expression profiles of *ice-2* and *ice-5* in *Bombyx mori* cells after H_2_O_2_ stimulation. Real-time PCR analyses were performed using total RNA from cells that were collected at regular intervals from 0 h to 6 h after H_2_O_2_ stimulation. The relative *ice-2* (F = 255.187; df = 7, 16; p = 0.0001) and *ice-5* (F = 102.894; df = 7, 16; p = 0.0001) expression levels as calculated by 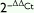 are shown for each group, and the bar charts (mean ± SE) represent three independent experiments with three replications. High quality figures are available online.

**Table 2:**
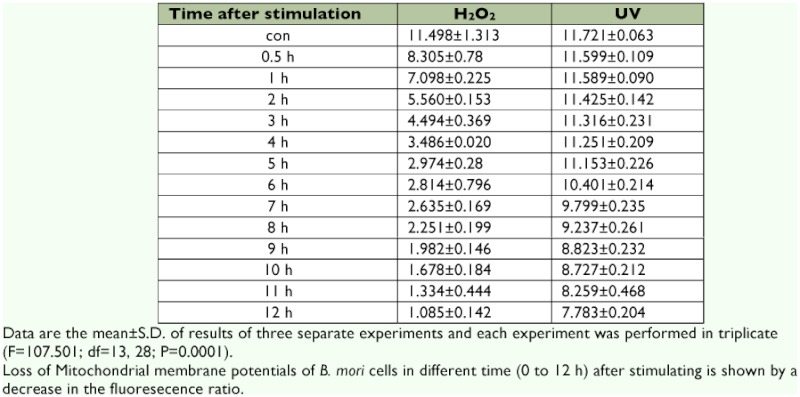
Change of the 590:530 fluorescence ratio of JC-1 dye after H_2_O_2_ and UV stimulation.

### Change in mitochondrial membrane potentials

*B. mori* cells were acutely exposed to 3 µM H_2_O_2_ and were tested at different times using the JC-1 assay. The results showed that during the first 5 h, the 590:530 fluorescence ratio of JC-1 dye declined sharper than that during the following 7 h, and the change could be omitted compared to the later change (Table 2). The red-green JC-1 fluorescence ratio started to decrease at 0.5 h after H_2_O_2_ stimulation. After dramatically declining, the red-green JC-1 fluorescence ratio tailed off steadily from 6 h to 12 h after-stimulation.

### Expression profiles of the *ice-2* and *ice-5* genes

The relative expression of mRNA of *ice-2* and *ice-5* of H_2_O_2_ stimulated *B. mori* cells was analyzed by quantitative real-time PCR. The *ice-2* gene was highly expressed at two time points, 0.5 and 5 h after H_2_O_2_ stimulation, while the expression level of *ice-5* peaked at 0.5, 3, and 5 h after H_2_O_2_ stimulation (Figure 3). In other times, however, very low levels of both *ice-2* and *ice-5* mRNAs were detected. The mRNA level of *ice-5* was higher than that of *ice-2* at the majority of time stages from 0 to 6 h, except for the 5 h time point.

### Comparisons between damage from H_2_O_2_ and UV irradiation

**Figure 4.**
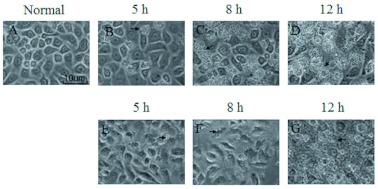
Comparison of morphology of UV and H_2_O_2_ treated *Bombyx mori* cells. The numbers at the top of the panels show the time stage of the *B. mori* cell culture after-stimulation. The black arrow indicates typical morphology of the cell in the relevant stage. (A) shows normal *B. mori* cells. (B–D) show UV treated cells. (E–G) show H_2_O_2_ treated cells. The photos were taken at 200× magnification. High quality figures are available online.

Although at 5 h post stimulation, the images of dying *B. mori* cells treated with H_2_O_2_ were distinct from UV treated cells, they both had similar appearances at 12 h (Figure 4). Apoptotic bodies could be found easily under a microscope at 200× magnification. Moreover H_2_O_2_ treated cells formed membrane vesicles at 9 h, while UV treated cells started to vesicluate at 5 h, when the response of the cells to the stimuli was first detected. Additionally, throughout the process, the change in the fluorescence ratio of H_2_O_2_ treated cells (10.413) was more obvious than that of the UV treated cells (4.938) (Table 2). In H_2_O_2_ treated cells, the fluorescence ratio declined at 0.5 h, but it declined at 6 h in UV treated cells (Table 2).

## Discussion

As previously reported, the decrease of mitochondrial membrane potential started at the very beginning of the treatment and preceded the morphological change of the cells. This implies that apoptosis induced by H_2_O_2_ might relate to the intrinsic apoptotic pathway via effects on the mitochondria. The peak levels of *ice-2* and *ice-5* were reached when the cellular morphology was still unchanged but the mitochondrial membrane potential had already changed considerably (Figures 2 and 3, Table 2), suggesting that the activation of *B. mori ice-2* and *ice-5* might be related to the release of cytochrome *c* from the mitochondria. Later, at 5 h after stimulation, changes in all the data were obvious. First, cell membranes were triggered to wrinkle, and cells became smaller than the ordinary cells. At the same time, the mitochondrial membrane potential steadily declined, beyond the dramatic decrease during the first 5 h. There was also another increase in the expression of *ice-2* and *ice-5*. In *Spodoptera frugiperda* cells, oxidant treatments resulted in the release of cytochrome *c* followed by the activation of caspase-3 (Sahdev et al. 2003). Therefore, *B. mori* ICEs might be regulated by H_2_O_2_, and related to the dysfunction of mitochondria, *ice-2* and *ice-5* may also be initiators associated with mitochondria initially, and then be effectors following the dysfunction of mitochondria in H_2_O_2_ induced apoptosis.

The fact that the genes of *ice-2* and *ice-5* were different from each other by just one exon implied that different mRNAs are present. This is likely related to the different patterns in their expression profiles. From 0 to 0.5 h after exposure to H_2_O_2_, while the level of *ice-2* increased from low to high, the level of *ice-5* increased from being undetectable to the highest level (Figure 3). Then, after expressing stable levels for a while, *ice-2* rose to its highest level, and *ice-5* reached its second peak, suggesting that *ice-5* may play a more active role in the early phase of H_2_O_2-_induced apoptosis than *ice-2*, and that they may have complementary functions, *ice-2* and *ice-5* might induce their own expression of in the later phases of apoptosis.

**Figure 5.**
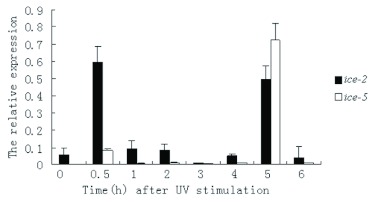
Expression profiles of *ice-2* and *ice-5* in *Bombyx mori* cells after UV stimulation (Wang et al. 2008). Real-time PCR analyses were performed using total RNA from cells that were collected at regular intervals from 0 h to 6 h after H_2_O_2_ stimulation. The relative *ice-2* and *ice-5* expression levels as calculated by 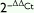 are shown for each group, and the bar charts (mean ± SE) represent three independent experiments with three replications. High quality figures are available online.

Based on the expression profiles, the levels of both *ice-2* and *ice-5* decreased significantly at 1 h after H_2_O_2_ stimulation, and the level of *ice-2* remained low from 1 to 4 h after H_2_O_2_ stimulation. In contrast, the level of *ice-5* fluctuated from low to a medium during this period. This was quite different from the profile of UV induced apoptosis (Figure 5). During UV induced apoptosis, from 1 to 4 h post treatment, *ice-5* was almost undetectable. This difference may have resulted in the changing morphology of *B. mori* cells at 5 h after stimulation. The unique expression patterns of *ice-2* and *ice-5* suggest that the single exon difference between them may be the reason for the unique role of *ice-5* in the apoptotic pathway.

In addition, the total reduction in fluorescence ratio of H_2_O_2_ treated cells is about 3 times more than the reduction in fluorescence ratio of UV treated cells. This suggests that H_2_O_2_-induced damage led to a more serious loss in the potential of the mitochondrial membrane (Table 2). This may have happened because UV irradiation damage to cells is only partly due to oxidative damage causing mitochondrion dysfunction (Kannan and Jain 2000). When the UV irradiation causes DNA mutation, DNA repair mechanisms might function to restore some mutations, so that both *ice-2* and *ice-5* were less active in UV stimulated cells.

In conclusion, *ice-2* and *ice-5* synchronal expression profiles indicate that activation of *ice-2* and *ice-5* may be related to mitochondrial dysfunction after H_2_O_2_-induced damage and that *ice-2* and *ice-5* might cooperate in the early phases of both H_2_O_2_ and UV induced apoptosis in a *B. mori* cell line. The comparison between relative expression profiles of H_2_O_2_ and UV-induced apoptosis suggests that the absence in *ice-2* of an 84bp exon that exists in *ice-5* might be the reason for lower activity of *ice-2* than of *ice-5* in the H_2_O_2_ induced apoptosis pathway. Because UV irradiation not only induces the generation of OH and H_2_O_2_ (Kannan and Jain 2000), but also can cause mutation of DNA, UV induced apoptosis is more complex than H_2_O_2_-induced apoptosis. This phenomenon would occur uniquely in UV irradiationinduced apoptosis and is a topic for further study.
